# Development and validation of a multivariable risk prediction model for serious infection in patients with psoriasis receiving systemic therapy

**DOI:** 10.1111/bjd.17421

**Published:** 2019-01-15

**Authors:** Z.Z.N. Yiu, C. Sorbe, M. Lunt, S.J. Rustenbach, L. Kühl, M. Augustin, K.J. Mason, D.M. Ashcroft, C.E.M. Griffiths, R.B. Warren, the BADBIR Study Group, Anthony D. Ormerod, Jonathan N.W.N. Barker, Ian Evans, Kathleen McElhone, Catherine H. Smith, Nick J. Reynolds, Ruth Murphy, Marilyn Benham, A. David Burden, Sagair Hussain, Brian Kirby, Linda Lawson, Caroline M. Owen

**Affiliations:** ^1^ Dermatology Centre Salford Royal NHS Foundation Trust The University of Manchester Manchester Academic Health Science Centre NIHR Manchester Biomedical Research Centre Manchester M13 9PT U.K; ^2^ Centre for Pharmacoepidemiology and Drug Safety School of Health Sciences The University of Manchester Manchester M13 9PT U.K; ^3^ IVDP – Institute for Health Services Research in Dermatology and Nursing University Medical Center Hamburg‐Eppendorf Hamburg Germany; ^4^ Arthritis Research U.K. Epidemiology Unit The University of Manchester Manchester M13 9PT U.K

## Abstract

**Background:**

Patients with psoriasis are often concerned about the risk of serious infection associated with systemic psoriasis treatments.

**Objectives:**

To develop and externally validate a prediction model for serious infection in patients with psoriasis within 1 year of starting systemic therapies.

**Methods:**

The risk prediction model was developed using the British Association of Dermatologists Biologic Interventions Register (BADBIR), and the German Psoriasis Registry PsoBest was used as the validation dataset. Model discrimination and calibration were assessed internally and externally using the *C*‐statistic, the calibration slope and the calibration in the large.

**Results:**

Overall 175 (1·7%) out of 10 033 participants from BADBIR and 41 (1·7%) out of 2423 participants from PsoBest developed a serious infection within 1 year of therapy initiation. Selected predictors in a multiple logistic regression model included nine baseline covariates, and starting infliximab was the strongest predictor. Evaluation of model performance showed a bootstrap optimism‐corrected *C*‐statistic of 0·64 [95% confidence interval (CI) 0·60–0·69], calibration in the large of 0·02 (95% CI −0·14 to 0·17) and a calibration slope of 0·88 (95% CI 0·70–1·07), while external validation performance was poor, with *C*‐statistic 0·52 (95% CI 0·42–0·62), calibration in the large 0·06 (95% CI −0·25 to 0·37) and calibration slope 0·36 (95% CI −0·24 to 0·97).

**Conclusions:**

We present the first results of the development of a multivariable prediction model. This model may help patients and dermatologists in the U.K. and the Republic of Ireland to identify modifiable risk factors and inform therapy choice in a shared decision‐making process.

## Abstract 2

**What's already known about this topic?**

- Patients and their clinicians are often concerned about the risk of serious infection associated with biological therapies for the treatment of psoriasis.
- However, there are no current tools available to estimate an individual's risk of serious infection when starting a systemic therapy.

**What does this study add?**

- This study found that the serious infection risk prediction model had good calibration and moderate discrimination
- The model included chronic obstructive pulmonary disease, alcohol intake, number of comorbidities and employment status, in addition to age, sex, Psoriasis Area and Severity Index, choice of starting therapy and body mass index.
- These are the first results of the multivariable prediction model, which may help patients and dermatologists in the U.K. and the Republic of Ireland to identify modifiable risk factors and inform therapy choice in a shared decision‐making process.

https://www.bjdonline.com/article/Development-and-validation-of-a-multivariable-risk-prediction-model-for-serious-infection-in-patients-with-psoriasis-receiving-systemic-therapy/

https://doi.org/10.1111/bjd.17664 available online

Given that the majority of systemic therapies for psoriasis suppress the immune system, patients and their clinicians are often concerned about an associated risk of serious infection. Patients with psoriasis often experience comorbidity and have lifestyle factors that themselves may be associated with a higher risk of serious infection than in the general population.^1^

A recent study found that in patients who do receive systemic therapies, 12% of patients practise intentional nonadherence, where patients decide not to follow the prescribed medication regimen.^2^ This may stem from concerns about potential negative effects of treatment such as the associated risk of infection or other serious adverse events. It could lead to a missed opportunity of achieving a good clinical outcome, in turn impacting on the achievable quality of life for the patient. Addressing patient concerns about the risk of serious infection associated with systemic therapies is, therefore, a vital aspect of any consultation prior to initiating such therapies. To our knowledge, there are no tools available to help patients with psoriasis or their clinicians to estimate the risk of serious infection when starting a systemic therapy.

Our objective was to develop a multivariable prediction model for serious infection within 1 year of systemic therapy initiation in patients with psoriasis. Within the European Network of Centres for Pharmacoepidemiology and Pharmacovigilance network of psoriasis registries (http://www.psonet.eu), we used a national, prospective psoriasis registry to develop and test the internal validity of the model [the British Association of Dermatologists Biologic Interventions Register (BADBIR)]. We then applied the model to a second national, prospective psoriasis registry (PsoBest, Germany) to test the external validity. Such a model would enable the dermatologist and patient to determine a personalized risk of first serious infection within 1 year of systemic therapy initiation.

## Patients and methods

### Data sources and study population

Data from BADBIR^3^ were used to develop the serious infection risk prediction model. To test whether this risk prediction model was generalizable to other populations, external validation using a cohort from the German Psoriasis Registry PsoBest^4^ was performed. The structures of these two registries are very similar.

### Development cohort (U.K. and Republic of Ireland)

BADBIR is a large, ongoing, prospective pharmacovigilance registry of patients with psoriasis. It was established in 2007 in the U.K. and Ireland to compare the safety of biological therapies against nonbiological systemic therapies. In total 153 secondary‐care dermatology centres in the U.K. and Ireland have contributed to the registry in this data snapshot. Patients with a Psoriasis Area and Severity Index (PASI) ≥ 10 and Dermatology Life Quality Index (DLQI) > 10 initiating a systemic biological or nonbiological medication are eligible for inclusion in the register. In England, the National Institute for Health and Care Excellence (NICE) recommends that all patients with psoriasis on biological therapies should be registered on BADBIR. Data are collected 6 monthly for the first 3 years, then annually thereafter. Detailed assessments at baseline and follow‐up have been performed. A data snapshot from February 2017 was used in the development dataset.

### Validation cohort (Germany)

The German Psoriasis Registry PsoBest is a national prospective patient registry established in 2008. In total 865 dermatology practices (*n *=* *797) and clinics (*n *=* *68) are registered in PsoBest from across Germany.^4^ Detailed assessments at baseline and follow‐up are also performed, similarly to those with BADBIR.^5^ A data snapshot from June 2017 was used as the validation dataset.

### Data analysis

Eligible criteria from both BADBIR and PsoBest include patients with chronic plaque psoriasis starting either a licensed biological therapy for psoriasis – for example etanercept (Enbrel), adalimumab (Humira), ustekinumab (Stelara) and infliximab (Remicade) – and patients starting acitretin, psoralen–ultraviolet A, ciclosporin, fumaric acid esters, methotrexate and hydroxycarbamide.

Patients in both cohorts contributed to the study if they had a follow‐up period of 1 year or more, or had < 1 year of follow‐up but developed one or more serious infections within the follow‐up period. Follow‐up was measured from the first prescribed dose of the first therapy while registered in either cohort.

In BADBIR we defined serious infection as any infection that was associated with or prolonged hospitalization, required intravenous antimicrobial therapy or led to death.^6^ A similar definition was used for serious infections in the PsoBest registry, but it was not possible to include the use of intravenous antimicrobial therapy in the definition. Four additional cases were included from BADBIR based only on the use of intravenous antimicrobial therapy. We limited the outcome to having had one or more serious infections within the first year. Prior to model development, we performed descriptive analysis of the baseline covariates in both cohorts.

### Model development

Baseline predictor covariates were identified a priori from consensus in the BADBIR research analysis group and a literature review.^7^ A two‐tier predictor selection process was performed. The first tier identified covariates that were highly likely important factors for the development of serious infections. These covariates were age, sex, PASI, choice of starting therapy and body mass index (BMI) at baseline. The nearest PASI score within 6 months of the date of therapy initiation was eligible for inclusion.

Regardless of the bivariate analysis results, these covariates were included in the final multiple logistic regression model. The second tier identified covariates that were probable important factors for the development of serious infections. These covariates were alcohol intake per week, cigarettes smoked per day, chronic obstructive pulmonary disease (COPD), asthma, depression, diabetes, hypertension, employment status, number of comorbidities, presence of psoriatic arthritis, total number of previous biological therapies, and total number of previous nonbiological systemic treatments. The number of comorbidities denoted the patient's self‐identified list of comorbidities. Employment status was divided into three categories as previously defined:^8^ (i) those working full time, part time or full time in the home, or who were students; (ii) those who were unemployed but seeking work or not working due to disability or ill health; and (iii) those who were retired.

The information for the predictors was captured from patient‐reported baseline questionnaires and clinician‐completed questionnaires. As we included only variables available to the clinicians at baseline prior to initiation of therapy, we did not include time‐varying factors such as switching of therapy or use of concomitant or combination systemic therapies. For this list of predictors a ratio of 10 events per predictor variable was achieved.

The outcome of serious infection within 1 year of treatment initiation was treated as a binary outcome, with values of 0 for no infection and 1 for at least one infection within 1 year. Bivariate logistic regression models were performed to obtain unadjusted odds ratios. The second‐tier potential predictors were assessed for inclusion using backward elimination in a logistic regression model, with a *P*‐value of 0·1 as the cut‐off. The equation for the prediction of serious infection was produced from the resulting multiple logistic regression model.

### Internal validation

The model's discriminative performance was assessed by the concordance statistic (*C*‐statistic, equivalent to the area under the receiver operating characteristic curve), representing the probability that the model would identify patients having had a serious infection in the 1 year as having a higher predicted risk than patients without such an event. The model calibration was assessed using the calibration slope and the calibration in the large (CITL). CITL measures whether the predicted prevalence was less than (CITL < 0) or greater than the observed prevalence (CITL > 0). The calibration slope primarily detects whether the model is overfitted (slope < 1).^9^ Internal validation was performed on the prediction model; the statistical details are presented in Appendix S1 (see Supporting Information).

### External validation

The final risk prediction model, after adjustment for overfitting, was applied to each participant in the external validation cohort. The performance of the model was similarly assessed by the *C*‐statistic (discrimination), the calibration slope and the CITL (calibration).

All analyses were performed using Stata version 15·1 (StataCorp, College Station, TX, U.S.A.). The study was conducted and reported according to the Transparent Reporting of a multivariate prediction model for Individual Prediction Or Diagnosis (TRIPOD) guidelines.^10^

### Ethical approval

BADBIR was approved in March 2007 by the National Health Service Research Ethics Committee North West England, reference 07/MRE08/9. All patients gave written informed consent for their participation in the registry. Approval for PsoBest was obtained in July 2007 from the ethics commission of the State Medical Association Hamburg (no. 2805).

## Results

**Figure 1:**
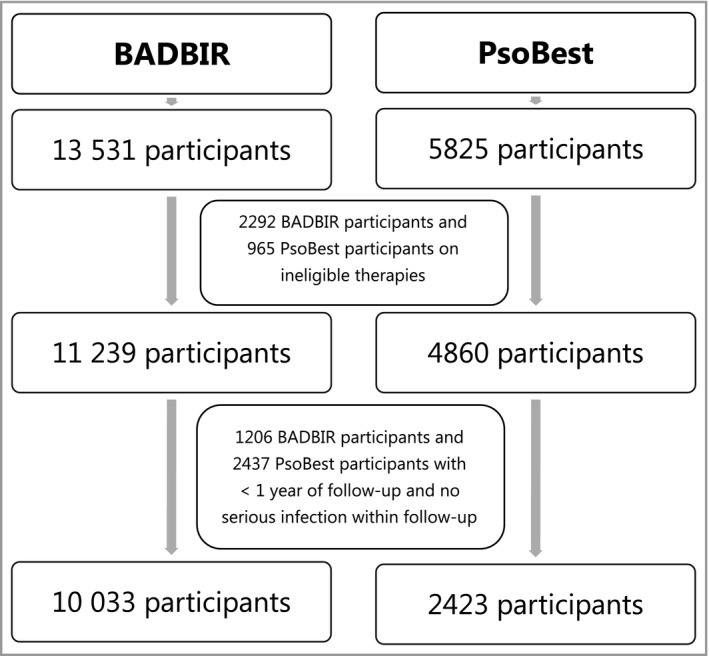
Flow diagram depicting the participant selection numbers of the development dataset (British Association of Dermatologists Biologic Interventions Register, BADBIR) and the validation dataset (PsoBest).

In the development cohort from BADBIR, 10 033 patients were eligible for inclusion. In the validation cohort from PsoBest, 2423 patients were eligible for inclusion (Fig. 1). Table S1(see Supporting Information) summarizes the background characteristics of the two registry populations. Broadly, patients from PsoBest were older and more likely to be current smokers, drink more alcohol, be currently working and have less comorbidity, but were less likely to have a BMI > 30 kg m^−2^ or depression. Regarding treatment, patients from PsoBest were more likely to receive nonbiological options and less likely to receive biological options.

### Model development, performance and validation

From BADBIR, 175 patients (1·7%) developed a serious infection within 1 year of starting systemic therapy. The location types of serious infections in both cohorts as coded by the High‐Level Term in the Medical Dictionary for Regulatory Activities are presented in Table 1, and the associations between the baseline covariates and serious infection in bivariate models are presented in Table S2 (see Supporting Information). In the multiple logistic regression backward selection model, the variables of COPD, alcohol intake, number of comorbidities and employment status were selected in addition to age, sex, PASI, choice of starting therapy and BMI (Table 2). The apparent and internal validation performance statistics of the final multivariable model are presented in Table 3.

**Table 1:** Location types of serious infections as coded by the MedDRA High‐Level Terms in BADBIR and the equivalent numbers in PsoBest

	BADBIR	PsoBest
Lower respiratory tract and lung infections	73 (0·7)	5 (0·2)
Urinary tract infections	32 (0·3)	< 5
Abdominal and gastrointestinal infections	15 (0·1)	9 (0·4)
Skin structures and soft‐tissue infections	12 (0·1)	< 5
Sepsis, bacteraemia, viraemia and fungaemia	11 (0·1)	< 5
Upper respiratory tract infections	11 (0·1)	5 (0·2)
Ear infections	8 (0·1)	< 5
Cardiac infections	6 (0·1)	0
Bone and joint infections	5 (0)	0
Female reproductive tract infections	< 5	< 5
Male reproductive tract infections	0	< 5
Hepatobiliary and spleen infections	< 5	< 5
Dental and oral soft‐tissue infections	0	< 5
Muscle and soft‐tissue infections	0	< 5
Eye and eyelid infections	0	< 5
Vascular infections	0	< 5

The data are the number (percentage proportion) of each type of serious infection out of all participants; *n*‐values < 5 are censored due to patient confidentiality. MedDRA, Medical Dictionary for Regulatory Activities; BADBIR, British Association of Dermatologists Biologic Interventions Register.

**Table 2:** Final multivariable prediction model for risk of serious infection 1 year after initiation of therapy in the development cohort (British Association of Dermatologists Biologic Interventions Register)

Variable	OR (95% CI)
Age	1·00 (0·98–1·01)
Female sex	1·35 (1·13–1·95)
Starting drug	
Nonbiological systemics	Reference
Etanercept	0·87 (0·54–1·40)
Infliximab	3·55 (2·03–6·21)
Adalimumab	1·10 (0·77–1·54)
Ustekinumab	1·30 (0·87–1·93)
PASI	1·01 (0·99–1·02)
Alcohol (units per week)	1·01 (1·00–1·02)
Number of comorbidities	1·08 (1·04–1·13)
COPD	1·78 (1·01–3·11)
Body mass index	1·01 (0·99–1·03)
Working status	
Working	Reference
Unemployed	1·42 (0·99–2·04)
Retired	2·05 (1·28–3·31)

PASI, Psoriasis Area and Severity Index; COPD, chronic obstructive pulmonary disease; OR, odds ratio; CI, confidence interval.

**Table 3:** Internal and external validation model diagnostics (95% confidence intervals)

	*C*‐statistic	Calibration in the large	Calibration slope
Apparent performance	0·68 (0·63–0·72)	0·006 (−0·15–0·16)	1·02 (0·84–1·20)
Internal validation test performance	0·66 (0·61–0·70)	0·009 (−0·14–0·16)	0·87 (0·69–1·04)
Average optimism	0·033	−0·009	0·13
Optimism‐corrected performance	0·64 (0·60–0·69)	0·015 (−0·14–0·17)	0·88 (0·70–1·07)
External validation performance	0·52 (0·42–0·62)	0·057 (−0·25–0·37)	0·36 (−0·24–0·97)

The optimism‐adjusted model discrimination gave a *C*‐statistic of 0·64 [95% confidence interval (CI) 0·60–0·69], and the optimism‐adjusted model calibration gave a CITL of 0·015 (95% CI −0·14 to 0·17) and a calibration slope of 0·88 (95% CI 0·70–1·07). A uniform shrinkage factor of 0·87 (the calibration slope of the test performance, Table 3) was applied to the beta‐coefficients of the multivariable model. The mean predicted probability of serious infection within 1 year of initiating therapy after adjustment for overfitting was 0·017 (SD 0·014, range 0·006–0·35).

As examples using the shrinked coefficients, if a 1% risk cut‐off was applied to identify serious infection, the sensitivity of the test would be 90·3% and the specificity 16·9%. If a 2% risk cut‐off was applied to identify serious infection, the sensitivity of the test would be 46·3% and the specificity would be 78·3%. The risk prediction equation for the log odds of the 1‐year serious infection risk is given in Appendix 2.

### External validation

From PsoBest, 41 patients (1·7%) developed a serious infection within 1 year of starting systemic therapy. The performance of the final risk prediction model in the PsoBest dataset gave a *C*‐statistic of 0·52 (95% CI 0·42–0·62), a CITL of 0·06 (95% CI −0·25 to 0·37) and a calibration slope of 0·36 (95% CI −0·24 to 0·96). The mean predicted risk of serious infection within 1 year after systemic therapy initiation was 0·016 (SD 0·011, range 0·006–0·18). Table S3 (see Supporting Information) shows low relatedness between the BADBIR and the PsoBest datasets. In an additional exploratory analysis, hypertension was selected in addition to the other covariates of the BADBIR prediction model as a strong predictor of 1‐year serious infection in the PsoBest cohort (Table S4; see Supporting Information). The *C*‐statistic of this exploratory model was 0·67 (95% CI 0·59–0·75).

## Discussion

We developed a multivariable model to predict the risk of serious infection within 1 year of starting a systemic therapy in patients with moderate‐to‐severe psoriasis. To our knowledge, this is the first study to use two large psoriasis treatment registries to develop and validate externally for serious infection after starting systemic therapy for psoriasis. However, the discriminative activity of the model after internal validation and adjustment for optimism was not perfect, with a *C*‐statistic of 0·64. At this stage, we feel that this is the first in several systematic steps in working towards a clinically applicable model for predicting serious infection in patients with psoriasis.

The strengths of this study are (i) the use of a large, national, prospective registry representative of patients with moderate‐to‐severe psoriasis in the real world to develop the algorithm and (ii) detailed data capture allowing inclusion of covariates that are clinically most relevant to the risk of infection for consideration in the model. The covariates included are readily available in a clinical setting, and the risk prediction score can be easily applied in the clinic.

The main limitation of the model was a *C*‐statistic below 0·70. Thus, it is likely that factors or interactions between variables that might be predictive of serious infection were not included. For example, previous history of a serious infection was not captured consistently, we do not measure adherence to medication, and geographical location may be influential in the risk of serious infection. Due to the high proportion of missing data, DLQI^11^ could not be included as a covariate. There may be residual confounding from covariates that were not known to be associated with the risk of serious infection, for example from nonmedical domains such as demographical factors, personal hygiene practices, predisposing work conditions and other lifestyle factors, and were therefore not included.

Another limitation is the low number of outcome events. As sample size in prediction models is dictated by the number of events per variable, the number of serious infection events (175) restricted the number of covariates that could be included in the model. This limits our ability to include other variables that could potentially improve the discrimination of the model.

We found statistically significant clinical covariates that were predictors for serious infection in patients receiving systemic therapies for psoriasis. Female patients had 35% higher odds of developing a serious infection than male patients. This result was unsurprising, given that we had previously reported that female participants in the BADBIR cohort had a 79% higher risk of discontinuing biological therapies due to an adverse event.^12^

The total number of comorbidities, a proxy for patient frailty, was also a significant predictor, and each additional comorbidity was associated with an 8% higher odds of serious infection. COPD was selected in the model as an important comorbidity, associated with an odds ratio of 1·78 of serious infection. This is because many patients are hospitalized for treatment of infective exacerbations of COPD. Alcohol intake was associated with a small but significant increase in the odds of serious infection, with each unit per week associated with an odds ratio of 1·01.

Although age was a covariate forced into the model, surprisingly this was not a statistically significant predictor of infection. This is likely due to the fact that working status, which is a proxy of functional status and overall health, was also included and adjusted for in the model, with retired participants associated with twice the odds of serious infection than participants who were working. The predictor with the highest strength of association with serious infection was treatment with infliximab. In separate time‐to‐event analyses, participants on infliximab in BADBIR^13^ and the Psoriasis Longitudinal Assessment and Registry, a large U.S.A.‐based psoriasis registry,^14^ also found that infliximab was associated with a higher risk of serious infection.

The BADBIR and PsoBest registry cohorts are substantially different from each other (Table S4; see Supporting Information). This difference is exemplified by the results of an additional exploratory analysis. We found that hypertension, which was not significant in the BADBIR cohort, was strongly predictive for serious infection in the PsoBest cohort. In addition, the odds ratios of working status, female sex, individual treatments and the number of comorbidities were also very different between the two models (Table S4).

There are several notable differences between the two registries. Firstly, there are differences in the proportions of patients on nonbiological systemic therapies and biological therapies (Table S1 ; see Supporting Information). Secondly, patients in BADBIR on nonbiologics are predominantly using methotrexate,^15^ while patients in PsoBest on nonbiologics are predominantly using fumaric acid esters.^16^ In addition, infliximab is only recommended to be used in the U.K. for patients with very severe psoriasis (PASI ≥ 20, DLQI > 18). This introduces confounding by indication that is not present in the PsoBest population.

Both the heterogeneity of outcome frequency and poor model transportability between the two different psoriasis registries are not surprising. A study by Psonet, a European collaboration of psoriasis registries, investigated the risk of serious infection in patients receiving tumour necrosis factor inhibitors with data from Italian (Psocare) and Spanish (BIOBADADERM) psoriasis registries and a healthcare database in Israel (Clalit Health Service database).^17^ That study showed marked heterogeneity in the infection rates between the individual countries (*I* ^2^ > 90%). The authors attributed this to variation in the definition of what constituted a ‘serious’ infection, which is also likely to have contributed to differences between BADBIR and PsoBest.

The propensity to admit a patient into the hospital, a key determinant of whether an infection is classed as ‘serious’, varies between countries, and it is notable that all of the BADBIR centres were from hospital clinics in secondary care, in contrast to the participation of predominately individual dermatology clinics and practices of primary care in PsoBest. This difference in healthcare systems may also introduce confounding by indication. For example, individual practices in primary care in Germany may be more reluctant to prescribe infliximab given that it needs to be administered in a hospital setting. It is possible that patients attending hospital clinics may be more likely to be admitted directly into the hospital if a serious infection was suspected compared with primary‐care clinics.

The risk prediction model would facilitate discussions between the clinician and the patient regarding risk factors for serious infection. As the interpretation of the risk of serious infection and decision regarding treatment choice may reflect the personal values of the patient, such a model could facilitate the shared decision‐making process. However, it is not possible to quantify the risk of serious infection precisely at the present stage of model development.

In conclusion, we have developed a preliminary prediction model for the risk of developing a serious infection within 1 year of initiation of systemic therapy in patients with psoriasis. We envisage that such an algorithm could help clinicians and the patient to choose the right starting therapy, discuss lifestyle choices and identify risk‐minimizing modifiable behaviours in shared decision making with their patients. Further development of the model, for example the inclusion of other predictors, and temporal validation using the U.K. and Ireland population of patients in BADBIR, is warranted after further data accrual.

## Supporting information


**Appendix S1** Supplementary methods.

**Table S1** Background characteristics of the development cohort (British Association of Dermatologists Biologic Interventions Register) and validation cohort (PsoBest).

**Table S2** Crude odds ratios from bivariate logistic regression models.

**Table S3** Assessment and measures of relatedness between the development and validation datasets.

**Table S4** Comparison of the final multivariable prediction model for risk of serious infection 1 year after initiation of therapy in the British Association of Dermatologists Biologic Interventions Register and an exploratory multiple logistic regression model with additionally selected covariates in PsoBest.

**Video S1** Author video.

## Appendix 1 Conflicts of interest and funding sources

Funding sources: BADBIR is coordinated by the University of Manchester and funded by the British Association of Dermatologists (BAD). The BAD receives income from Celgene, Hexal AG, Almirall, Pfizer, Janssen‐Cilag, AbbVie, Novartis, Samsung Bioepis and Eli Lilly for providing pharmacovigilance services. This income finances a separate contract between the BAD and the University of Manchester, who coordinate BADBIR. All decisions concerning analysis, interpretation and publication are made independently of any industry contribution. All relevant information regarding serious adverse events mentioned in this publication has been reported to the appropriate company as per the contractual agreements and standard operating procedures. PsoBest is coordinated by CVderm (the German Center for Health Services Research in Dermatology) of the Institute for Health Services Research in Dermatology and Nursing at the University Medical Center Hamburg‐Eppendorf. PsoBest is supported by AbbVie Deutschland GmbH, Almirall Hermal GmbH, Biogen GmbH, Celgene GmbH, Hexal AG, Janssen‐Cilag GmbH, LEO Pharma GmbH, Lilly Deutschland GmbH & Co. KG and Pfizer Deutschland GmbH. All decisions concerning analysis, interpretation and publication are made independently of any industry contribution. Z.Z.N.Y. is funded by a National Institute for Health Research (NIHR) Doctoral Research Fellowship (no. DRF‐2015‐08‐089). BADBIR acknowledges the support of the NIHR through the clinical research networks and the contribution made by clinical research associates who continue to facilitate recruitment into the registry. The research was supported by the NIHR Manchester Biomedical Research Centre. The views expressed are those of the authors and not necessarily those of the National Health Service, the NIHR or the Department of Health. C.E.M.G. is an NIHR Senior Investigator. C.E.M.G., D.M.A. and R.B.W. are funded in part by the Medical Research Council (MR/L011808/1).

Conflicts of interest: C.E.M.G. is an NIHR Senior Investigator and reports grants from the NIHR, during the conduct of the study; grants and personal fees from GSK, AbbVie, Lilly, Novartis, Pfizer, Janssen, LEO and Celgene; grants from Sandoz; personal fees from Sun Pharmaceuticals, UCB Pharma and Almirall; and grants and personal fees from outside the submitted work. D.M.A. has received research grants from AbbVie and LEO Foundation. K.J.M. has received honoraria from Janssen‐Cilag and Eli Lilly. R.B.W. has received research grants from AbbVie, Pfizer, Novartis and LEO; and reports personal fees from AbbVie, Amgen, Boehringer Ingelheim Pharma, Celgene, Janssen‐Cilag, LEO, Lilly, Novartis, Pfizer and Xenoport outside the submitted work. Z.Z.N.Y. has received nonfinancial support from Novartis outside the submitted work. M.A. has served as a consultant and/or paid speaker for, and/or has received research grants and/or honoraria for consulting and/or scientific lectures for, and/or has received travel expenses reimbursed by, and/or has participated in clinical trials sponsored by companies that manufacture drugs used for the treatment of psoriasis including AbbVie, Almirall, Amgen, Biogen (Biogen Idec), Boehringer Ingelheim, Celgene, Centocor, Eli Lilly, Galderma, Janssen‐Cilag, LEO, Medac, MSD, Mundipharma, Novartis, Pfizer, Sandoz and Xenoport. C.S., L.K. and S.J.R. are employees of University Medical Center Hamburg‐Eppendorf. M.L. has no conflicts of interests to disclose.

## Appendix 2 Risk prediction model

The risk score from a logistic regression model to predict serious infection within 1 year of initiation of systemic therapy for psoriasis:

Log odds of serious infection within 1 year =−4·985364 − (0·0042559 × age) + (0·3036825 × female sex) + (0·0062117 × PASI) + (0·0084999 × units of alcohol per week) + (0·0809034 × number of comorbidities)+ (0·5743102 × COPD) + (0·0098085 × BMI) + (0·354117 × unemployed or not currently working)+ (0·7202485 × retired) − (0·140139 × etanercept) + (1·267439 × infliximab) + (0·0975531 × adalimumab) + (0·2599529 × ustekinumab).
