# Supplementary material for: Development and validation of a multivariable risk prediction model for serious infection in patients with psoriasis receiving systemic therapy
Source: Br J Dermatol. 2019 Jan 15;180(4):894–901. doi: 10.1111/bjd.17421 (PMC6850093; doi:10.1111/bjd.17421)
Supplement: Supplementary file 1 — Appendix S1 Supplementary methods. [file BJD-180-894-s001.docx]

***Supplementary Materials***

***Methods***

*Model development*

Continuous variables were tested for non-linearity. Where non-linearity was present, fractional polynomial transformation of continuous predictors were performed using the mfpmi command in Stata. This command uses a closed test procedure to determine whether a continuous predictor be included or would best be fitted using fractional polynomials or in a linear form using weighted likelihood ratio tests, with a stacked and weighted approach to take into account the multiple imputed datasets.

*Internal validation*

Overfitting, where the prediction model is overly complex and fitted to the random noise of the data rather than the underlying associations between the covariates and the outcome, results in predictions that are too extreme. Internal validation techniques help to estimate and adjust for the extent of optimism introduced in the model development process. Bootstrapping is a statistical technique of resampling with replacement from the original sample to introduce a random element, mimicking the process of sampling from the underlying population. Multiple imputation using chained equation approach based on all candidate predictors was used to create 20 imputed datasets. 200 bootstrap samples were produced from the development cohort. The model development process was repeated, including imputation for missing data and variable selection, in each bootstrap sample. Within each bootstrapped sample, a prediction model was developed and applied both to the original data (test performance) and the bootstrapped sample (apparent performance) to obtain average measures of optimism, the difference between the test and apparent performances, for the C-statistic, the calibration slope and the calibration-in-the-large across all the models. The average test calibration slope was applied as the uniform shrinkage factor to the original β coefficients to adjust for overfitting, and the intercept was re-estimated based on the shrunken β coefficients to produce the final model.

*Model validation*

Assessment of relatedness, which is a measure of the similarity of the development and validation cohorts, was performed. Discrimination was assessed by the C-statistic from the “membership model”, where a binary variable for the development sample against the validation sample was the dependent variable. A larger C-statistic indicates weak relatedness, while a smaller C-statistic indicates a strong relatedness. Calibration was assessed using the ratio of the standard deviation of the linear predictors, and the mean difference in linear predictors of the two samples. These performance measures compare the covariance and the mean of the variables selected in the final model, and further estimates the relatedness of the two cohorts. To gain a better understanding of the differences resulting in low model transportability an additional exploratory multiple logistic regression model was applied to the PsoBest cohort. The covariates selected in the BADBIR model were forced into the PsoBest model, but additional covariates available in PsoBest including metabolic syndrome and having had a previous infection were also tested for inclusion using backward elimination (p= 0.1).
