# Supplementary material for: Development and validation of a multivariable risk prediction model for serious infection in patients with psoriasis receiving systemic therapy
Source: Br J Dermatol. 2019 Jan 15;180(4):894–901. doi: 10.1111/bjd.17421 (PMC6850093; doi:10.1111/bjd.17421)
Supplement: Supplementary file 2 — Table S1 Background characteristics of the development cohort (British Association of Dermatologists Biologic Interventions Register) and validation cohort (PsoBest). [file BJD-180-894-s002.docx]

**Table S1** Background characteristics of the development cohort (BADBIR) and validation cohort (PsoBest)

| Variable – mean (SD) for continuous variables, n (%) for categorical variables | Development cohort (n=10033) | Validation cohort (n=2423) |
| --- | --- | --- |
| Age (years) | 45.0 (13.3) | 48.0 (14.0) |
| Female sex | 4154 (41.4) | 979 (40.4) |
| Disease duration (years) | 21.3 (12.8) | 18.2 (14.4) |
| PASI | 15.8 (7.9) | 14.3 (9.6) |
| Missing PASI | 1943 (19.4) | 42 (1.7) |
| Total number of comorbidities | 1.8 (2.2) | 1.1 (1.6) |
| Psoriatic arthritis | 1747 (17.4) | 480 (19.8) |
| Body mass index (kg/m^2^) | | |
| <18.5 | 96 (1.0) | 27 (1.1) |
| 18.5 – 24.9 | 1765 (17.6) | 653 (27.2) |
| 25.0 – 29.9 | 3018 (30.1) | 923 (38.4) |
| 30.0 – 34.9 | 2266 (22.6) | 504 (21.0) |
| ≥35.0 | 2106 (21.0) | 297 (12.4) |
| Missing BMI | 782 (7.8) | 19 (0.0) |
| Alcohol (units/week) | 8.1 (13.6) | 10.0 (7.6) |
| Missing alcohol intake | 1270 (12.7) | 73 (3.0) |
| Smoking status | | |
| Never smoked | 2634 (26.3) | 484 (21.4) |
| Previous smoker | 3114 (31.0) | 791 (35.0) |
| Current smoker | 1531 (15.3) | 982 (43.5) |
| Employment status | | |
| Working full-time | 6403 (63.8) | 1686 (73.1) |
| Unemployed but seeking work | 1424 (14.2) | 190 (8.2) |
| Retired | 964 (9.6) | 431 (18.7) |
| Missing employment status | 1242 (12.4) | 116 (4.8) |
| Hypertension | 2460 (24.5) | 666 (27.5) |
| Previous TB | 148 (1.5) | 6 (0.3) |
| Diabetes | 869 (8.7) | 231 (9.5) |
| Dyslipidaemia | 1142 (11.4) | 212 (8.8) |
| Asthma | 1098 (10.9) | 83 (3.4) |
| COPD | 201 (2.0) | 59 (2.4) |
| Depression | 2085 (20.8) | 179 (7.4) |
| Chronic renal disease | 270 (2.7) | 38 (1.6) |
| Total previous biologic treatments | 0.2 (0.5) | 0.2 (0.6) |
| Total previous systemic non-biologic treatments | 1.5 (1.2) | 1.0 (1.1) |
| Total previous systemic treatments | 1.7 (1.4) | 1.2 (1.5) |
| Starting therapy | | |
| Non-biologic | 3621 (32.5) | 1606 (66.3) |
| Etanercept | 1511 (13.6) | 184 (7.6) |
| Infliximab | 206 (1.9) | 70 (2.9) |
| Adalimumab | 4105 (36.8) | 347 (14.3) |
| Ustekinumab | 1701 (15.3) | 216 (8.9) |
| Serious infection in the 1^st^ year | 175 (1.7%) | 41 (1.7%) |
