# Supplementary material for: Development and validation of a multivariable risk prediction model for serious infection in patients with psoriasis receiving systemic therapy
Source: Br J Dermatol. 2019 Jan 15;180(4):894–901. doi: 10.1111/bjd.17421 (PMC6850093; doi:10.1111/bjd.17421)
Supplement: Supplementary file 3 — Table S2 Crude odds ratios from bivariate logistic regression models. [file BJD-180-894-s003.docx]

**Table S2** Crude odds ratios from bivariate logistic regression models

| Variable | Unadjusted OR (95% CI) |
| --- | --- |
| Age (years) | 1.02 (1.01–1.03)* |
| Female gender | 1.55 (1.15–2.08)* |
| Disease duration (years) | 1.01 (1.00–1.03) |
| PASI | 1.02 (1.00–1.04)* |
| Total number of comorbidities | 1.15 (1.11–1.19)* |
| Psoriatic arthritis | 1.32 (0.92–1.90) |
| Body mass index (kg/m^2^) | 1.02 (1.00–1.04)* |
| Alcohol (units/day) | 1.01 (1.00–1.02)* |
| Smoking status | |
| *Never smoked* | Ref |
| *Previous smoker* | 1.27 (0.85–1.91) |
| *Current smoker* | 1.08 (0.70–1.65) |
| Employment status | |
| *Working full-time* | Ref |
| *Unemployed but seeking work* | 1.97 (1.33–2.93)* |
| *Retired* | 2.87 (1.92–4.28)* |
| Hypertension | 1.71 (1.25–2.33)* |
| Previous TB | 0.77 (0.19–3.13) |
| Diabetes | 1.78 (1.16–2.73)* |
| Dyslipidaemia | 1.37 (0.90–2.08) |
| Asthma | 1.70 (1.14–2.54)* |
| COPD | 4.13 (2.30–7.40)* |
| Depression | 1.72 (1.24–2.38)* |
| Chronic renal disease | 1.99 (1.01–3.94)* |
| Total previous biologic treatments | 1.36 (1.08–1.71)* |
| Total previous systemic non-biologic treatments | 1.04 (0.92–1.18) |
| Total previous systemic treatments | 1.08 (0.98–1.20) |
| Starting therapy | |
| *Non-biologic* | Ref |
| *Etanercept* | 0.89 (0.52–1.52) |
| *Infliximab* | 4.99 (2.69–9.26)* |
| *Adalimumab* | 1.13 (0.77–1.66) |
| *Ustekinumab* | 1.55 (0.99–2.44) |

*p<0.05
