# Supplementary material for: Development and validation of a multivariable risk prediction model for serious infection in patients with psoriasis receiving systemic therapy
Source: Br J Dermatol. 2019 Jan 15;180(4):894–901. doi: 10.1111/bjd.17421 (PMC6850093; doi:10.1111/bjd.17421)
Supplement: Supplementary file 4 — Table S3 Assessment and measures of relatedness between the development and validation datasets. [file BJD-180-894-s004.docx]

**Table S3** Assessment and measures of relatedness between the development and validation datasets

| Difference in mean of linear predictor | −0.075 |
| --- | --- |
| Ratio of linear predictor standard deviation | 0.975 |
| *C*-statistic from membership model^a^ | 0.767 (0.756–0.777) |

The *C*-statistic measuring relatedness of the development and validation cohorts is high at 0.77. This indicates that the two cohorts are substantially different from each other, and therefore the external validation performance is likely to measure model transportability (performance of the model in new samples from different but related populations) rather than model reproducibility (performance of the model in new samples from the same target population). The small difference in mean of linear predictors (−0.075) indicates that the mean values of the covariates were, however, similar between two cohorts. The ratio of the standard deviations of linear predictors (0.97) between the cohorts is close to 1, indicating that there is little difference in the variability of the covariates between the two cohorts. ^a^Debray TP, Vergouwe Y, Koffijberg H *et al.* A new framework to enhance the interpretation of external validation studies of clinical prediction models. *J Clin Epidemiol* 2015; **68**:279–89.
