# Supplementary material for: Development and validation of a multivariable risk prediction model for serious infection in patients with psoriasis receiving systemic therapy
Source: Br J Dermatol. 2019 Jan 15;180(4):894–901. doi: 10.1111/bjd.17421 (PMC6850093; doi:10.1111/bjd.17421)
Supplement: Supplementary file 5 — Table S4 Comparison of the final multivariable prediction model for risk of serious infection 1 year after initiation of therapy in the British Association of Dermatologists Biologic Interventions Register and an exploratory multiple logistic regression model with additionally selected covariates in PsoBest. [file BJD-180-894-s005.docx]

**Table S4** Comparison of the final multivariable prediction model for risk of serious infection one year after initiation of therapy in BADBIR and an exploratory multiple logistic regression model with additionally selected covariates in PsoBest.

| Variable | OR (95% CI) in BADBIR | OR (95% CI) in PsoBest |
| --- | --- | --- |
| Age | 1.00 (0.98,1.01) | 1.00 (0.97, 1.03) |
| Female gender | 1.35 (1.13,1.95) | 0.74 (0.41, 1.55) |
| Starting drug | | |
| Non-biologic systemics | Ref | Ref |
| Etanercept | 0.87 (0.54,1.40) | 1.20 (0.35, 4.08) |
| Infliximab | 3.55 (2.03,6.21) | 1.88 (0.42, 8.38) |
| Adalimumab | 1.10 (0.77,1.54) | 1.94 (0.91, 4.15) |
| Ustekinumab | 1.30 (0.87,1.93) | 0.88 (0.26, 3.01) |
| PASI | 1.01 (0.99,1.02) | 0.97 (0.94, 1.01) |
| Alcohol (units per week) | 1.01 (1.00,1.02) | 0.99 (0.95, 1.04) |
| Number of comorbidities | 1.08 (1.04,1.13) | 0.81 (0.58, 1.12) |
| COPD | 1.78 (1.01,3.11) | 3.91 (1.03, 14.79) |
| BMI | 1.01 (0.99,1.03) | 1.06 (1.01, 1.11) |
| Working status | | |
| Working | Ref | Ref |
| Unemployed | 1.42 (0.99,2.04) | 0.90 (0.26, 3.12) |
| Retired | 2.05 (1.28,3.31) | 1.17 (0.41, 3.32) |
| Additional variables significant in PsoBest | | |
| Hypertension | / | 2.31 (0.98, 5.43) |
